# Effects of massage therapy of asthmatic children on the anxiety level of mothers

**Published:** 2010

**Authors:** Zohreh Ghazavi, Mahboubeh Namnabati, Jamal Faghihinia, Mohsen Mirbod, Parvin Ghalriz, Afsaneh Nekuie, Nasrin Fanian

**Affiliations:** * Department of Pediatrics Nursing, School of Nursing and Midwifery, Isfahan University of medical sciences, Isfahan, Iran; ** Assistant Professor, Department of Pediatrics, of Medicine, Isfahan University of medical sciences, Isfahan, Iran; *** Department of Physiotherapy, School of Rehabilitation, Isfahan University of Medical Sciences, Isfahan, Iran; **** Department of Health Nursing, School of Nursing and Midwifery, Isfahan University of Medical Sciences, Isfahan, Iran; ***** Hajar Hospital, Shahrekord University of Medical Sciences, Shahrekord, Iran; ****** Nour Hospital, Isfahan University of Medical Sciences, Isfahan, Iran

**Keywords:** Asthma, anxiety, children, massage therapy, mothers

## Abstract

**BACKGROUND:**

Asthma is the most common chronic disease of childhood and its prevalence is increasing all over the world. Asthma influences on many aspects of family daily life. Health care of children with chronic asthma can have deep impact on health and welfare of the family members. Studies showed a relation between the life quality of children suffering from asthma and the anxiety level of parents. These parents are looking for ways to confront with their stress, to reduce their anxiety in encountering with their asthmatic children, and to improve their performance. This research was accomplished with the aim of determining the influence of massage therapy on anxiety level of mothers with asthmatic children.

**METHODS:**

This was a quasi-experimental study with two groups and a pretest and posttest design. The samples of research were 60 mothers of 5-14 year-old asthmatic children who were under treatment in medical centers of Isfahan. They were randomly divided into two groups of control and massage therapy by convenience sampling method. The data were collected by standard Spielberger questionnaire. Mothers of massage group were trained to massage head, neck, face, shoulder, hand, leg, and back of their children every night before bedtime for one month while there was no intervention for the control group during this month except the standard treatment. In both groups, the Spielberger standard questionnaire was filled by mothers. The data were analyzed by descriptive analysis, independent t-test, paired-t test, and chi-square test.

**RESULTS:**

The results showed no significant difference in mean anxiety level between the two groups before the intervention but there was a significant difference between them after intervention (p < 0.03). Also, there was a significant difference in mean level of anxiety score of mothers before and after the intervention in massage group (p < 0.001).

**CONCLUSIONS:**

The anxiety level of mothers can be reduced by effective utilization of daily child massage therapy and giving an active role to the mothers in caring and treating the child. Daily massage helped mothers to have more sense of participation in caring their children and as a non-pharmacological method can be accompanied with pharmacological methods.

Many disorders in children cause chronic diseases. One of them is asthma which puts families in a sever crisis. Asthma is one of the most prevalent chronic diseases of childhood which is expected to be doubled all over the world by 2020.^1^,^2^ Primary data and statistics showed the prevalence rate of asthma in 10 to 15 percents of Iranian children and teenagers.^3^ This chronic disease has different effects on family performance and life.^2^ Sawyer study indicated a relationship between the quality of life of asthmatic children and the anxiety level of parents.^4^ Researchers found out that stress may have considerable effects on disease progression. Also, there is a relation between problems of children care and progressions of asthma in early childhood.

The risk of asthma attacks increased after stressful weeks. In children with stressful families, the probability of asthma progression is three to four times more common compared to normal families.^5^ some nurses believe that the most of the parents of asthmatic children think there is nothing more panicking than seeing their children not able to breathe normally. They feel hopeless in helping their children and this increases worries of children. Nurses also expressed that parents ask: “Cannot we do anything else? Is there anything we can do?” when they see their children striving to breath. The best answer to these questions is: “you can massage your child”.^6^ Massage therapy by parents can be a way for improving life quality and it is expected to have psychological effects on mothers.5 Parents can have an active role in health progression of child by doing massage therapy and also reduce their own stress. Lotta et al showed that parents of asthmatic children looking for ways to confront with stress, reduce anxiety about the asthmatic child, and increase their performance in encountering with asthmatic children.^7^ Many studies indicated the effects of massage therapy of children with chronic diseases on the anxiety level of parents. While Field et al showed that massage is an effective intervention in treating asthma attacks and improving the lung function of asthmatic children but has no effect on anxiety level of parents after one month.^8^ Given the fact that asthma is a common and chronic disease, especially in Iran, and that the children adaptation to the disease (short and long term) depends on how the families accept the disease, child and family support is a nursing duty. Besides, asthma managing programs can reduce the costs and increase the life quality of child and family and can provide a normal life for children.

Considering the fact that unlike other developed countries, no study has been accomplished in this regard in Iran, this research was done to determine the effects of massage therapy on anxiety level of mothers of asthmatic children, who have referred to the medical centers of Isfahan in 2005-2006. With hope that, these results can be presented as a scientific solution for reducing anxiety level of mothers of asthmatic children.

## Methods

This quasiexperimental study was accomplished by pretest and posttest design in two groups. The study population included mothers of children suffering from asthma who had inclusion criteria for the research. These criteria included mothers whose children were 5-14 years old and their mild to moderate asthma was definite according to the diagnosis of the physician. These children had not any other chronic diseases except asthma. Children who were selected had not any prohibited massage therapy (such as edema of the massage area, damaged tissue, cuts, burns, infectious rush, eye contact lens, taking anticoagulant drugs, and fracture on massage area), had biologic parents and had not any kind of diseases (cystic fibrosis and heart disease), and also were volunteer to participate in the study. Exclusion criteria were failure in follow-up the treatment for any reason, making new changes in drug treatment programs of asthma, uncooperative mother or child in doing the massage, mothers who had acute problems (death of a family member, getting divorce, and disease leading to hospitalization).

The participants in this study were 60 cases (massage and control groups) with 95% confidence coefficient and 80% power test. Sampling was accomplished by convenience method among the children referred to Asthma Clinic of Alzahra hospital and the pediatric clinic in 2005 for one year. The data were gathered by demographic questionnaire (interview and folder) and a twenty-item standard Spielberger Situational Anxiety Questionnaire that was given to all mothers to fill out just before starting the massage. Upon completion of an interview and describing the research objectives, qualified participants were asked to sign the consent forms.

Then, a meeting was hold with massage group (mothers and their children) during which the research objectives, the work performance, and the number of the sessions were explained. Later the participants of massage group were introduced to the pertinent physician’s office in groups of 1-2 persons where they were taught the massage techniques like kneading and stroking by one of the researchers. Upon completion of teaching, the participants were given a training video CD at the last session when possible questions were answered and some corrections were done in their misunderstandings. It was assured in this way that the mothers of massage group could massage their children’s head, neck, face, shoulders, hands, and feet for a month in kneading and stroking methods. But in control group, there was no intervention except receiving the treatment. One of the researchers made a phone call to the participants to check their status and make sure that the massage therapy was done twice a week. The researchers gave their phone numbers to the participants for possible questions they might have. In order to figure out the impact of massage therapy on the anxiety levels of mothers, the mothers of the both groups were asked to fill out the Spielberger Standard Questionnaire one month after beginning of the study. Cronbach’s alpha coefficient of 91% was confirmed the reliability of the Standard Questionnaire. In order to analyze the data, independent t-test, paired t-test and chi-square test were used in SPSS software.

The ethical committee of Isfahan University of Medical Sciences approved the study.

## Results

The majority of participants in this study were aged between 30-39 years old (55%), the age range of massage group was between 31-70 years, and was between 32-46 years in the control group. There was no significant difference between the two groups in this regard.

Based on obtained data, the majorities of the mothers were housekeepers (86.7%) and had the degree-level of diploma (63.3%). Distribution of educational level of mothers was similar in the two groups. The results showed no significant difference between the average age of children, the average duration of asthma and asthma severity between the two groups.

The independent t-test showed no significant difference in mean anxiety scores of mothers between the two groups before massaging. But, after doing the massage therapy, the mean anxiety score of mothers between the two groups had a significant difference (p < 0.03). Paired t-test showed a significant difference in mean anxiety score of mothers in massage group before and after the massage therapy (p < 0.001) but in the control group no significant difference was found (Table 1).

**Table 1:** Comparison of the mean anxiety scores of asthma children’s mothers before and after intervention in both control and massage group

Anxiety level	Massage group Mean (SD)	Control group Mean (SD)	P value
Before intervention	47.33(9.70)	48.06(13.95)	0.81
After intervention	40.9(9.98)	46.66(13.52)	0.03
P value	< 0.001	0.43	

## Discussion

The results indicated a significant difference between the mean anxiety score of mothers after the massage therapy intervention. Rydstorm et al believed that the families with asthmatic children have more anxiety and turbulent behavior patterns than families of children with other chronic diseases like diabetes mellitus.^10^ Hernandez et al also believed that chronic disease is a potential stressor (physiologic distress) for children and their families.^11^

The findings showed that involving the mothers in taking care of their children reduces their anxiety levels. Salvo expressed that the benefits of massage therapy are to reduce the parents stress in communicating with their children.^11^ Barlow et al revealed that the parents might expose to the physiologic distress with disabilities and lose their confidence to their abilities. Increasing the self-efficiency and the feeling of involvement in taking care of children are the ways for strengthen parents physiological wellbeing.^12^ Dugas also believed that massage therapy was an excellent method in increasing the participation rate of family members because with massaging of the back, hands or feet they are actively participating in patient treatment.^13^ Barlow et al indicated that the massage done by mothers caused a reduction in their anxiety levels.^14^ Also, the results of the study in which the impact of massage therapy on the anxiety level of the mothers was evaluated, showed a significant reduction in the anxiety level of the parents of children with atopic dermatitis in massage group.^15^ In the current study, a reduction in the anxiety level of the mothers was also seen. But the study of Field et al did not confirm our results. Of course, Field et al study findings showed the impact of massage therapy of asthmatic children on the anxiety level of the parents at the first day.^8^

This study also showed a significant difference between the anxiety scores of the massage group before and after the massaging. In this regard, in Powell et al study, which had an educational program and supported parents with cerebral palsy children, parents had reported that during the massaging their children they gained better feelings.^16^ In another study, the researchers also showed that the massaging by parents had significant effects on depression and parental abilities in children care.^17^ Hernandez et al also showed a significant reduction in the anxiety level of the mothers compared with their previous status based on the study which was about the impact of massage therapy on children with cystic fibrosis.^10^ Glover et al study findings indicated that parents depression in massage group improved compared with that in the control group.^18^ In the current study also, a significant reduction was observed in the anxiety levels of mothers in massage group compared with the control group.

This research showed no significant difference in anxiety levels of mothers in control group before and after the study. Some studies indicated that the parents of children with chronic diseases undertake the responsibility of the primary care most of the time. For this type of families, making a balance between spending time and complex treatment commands with other aspects of family life, job conditions, and leisure activities is considered as a major issue and concern.^10^ Field et al compared the difference between giving and receiving the massage, and made a conclusion that giving the massage has more positive effects.^19^ Lowdermilk et al believed that doing the massage for parents or care providers has some benefits too. As children get dependent to the parents, the parents would get dependent as well, and more important point is that doing the massage helps parents to have less anxiety levels while giving the massage.^20^

In the present study, in the massage group a significant reduction was seen in the anxiety level of the parents. It also indicated that parent’s involvement in their own child care leads to reduction of the anxiety levels of the mothers. The average score of the anxiety showed a significant difference between the two groups. It is said that massaging the child helps parent experience a lower anxiety level.^21^ Field et al study showed that in the group that mothers had given massage to their children twenty minutes before going to bed for 30 days, a significant reduction was seen in the anxiety and depression levels of the parents.^22^ Field et al study also indicated a significant reduction in anxiety levels of parents of children with diabetes in massage group compared with relaxation group.^23^

In this study, in group of mothers who used the standard method of massage for their children, a significant reduction was observed in the anxiety levels. The findings of this study showed that massage therapy could reduce the anxiety levels of mothers with asthmatic children. This massage treatment could also be considered as an effective way for improving mothers psychological well being. Moreover, daily massaging helps mothers feel more sense of involvement in their children care. Besides, enabling mothers to participate more fully in the treatment of asthmatic children can protect them from the risk of psychological problems. Therefore, giving the active role to the mothers in their children treatment may decrease the anxiety level; particularly, in this research children and mothers themselves liked the method. In order to find out more effects of massage therapy, further researches are needed. It is recommended that the impacts of massage therapy on the anxiety levels of mothers of asthmatic children be pursued on a longer time.

The authors declare no conflict of interest in this study.
